# Annotations

**Published:** 1906-10-13

**Authors:** 


### ANNOTATIONS.

### Pharmaceutical Chemistry and Therapeutics.

In this busy age the arts of the advertiser oppress, and sometimes circumvent, the medical practitioner even as they do other citizens. Especially, perhaps, are commercial methods applied to the exploitation of new remedies, and this to such an extent that many practitioners, wearied with the deluge of circulars and protests, consign them indiscriminately to the waste-paper basket. Yet, without question, experimental and research work in the pharmaceutical laboratory have in recent years added a number of valuable remedies to the physician's equipment. The task is to distinguish the sheep from the goats, for universal rejection is only one degree better than universal acceptance and adoption. Considerable aid in this direction can be obtained from the study of the volume of reports issued by E. Merck, of Darmstadt. This is not a mere trade catalogue produced for the purpose of advertising the products of the firm, and were it so it would, of course, merit no notice in this column. On the contrary, it is a carefully compiled scientific record of observations made by independent authorities both in the laboratory and in the ordinary course of clinical work. Results, whether favourable or otherwise from the therapeutic point of view, are duly chronicled, and the details both of the clinical facts and the methods of treatment are fully described, thus placing the practitioner in a position to judge for himself whether in an individual case under his own care he is justified in adopting any of the proposed plans. Altogether, as a reference book for the purpose of learning what contributions pharmaceutical chemistry is offering to practical therapeutics, the volume of reports is of great interest and importance. In its own line it is practically without a rival, and its merits entitle it to the most careful attention. It may be obtained by application to 16 Jewry Street, E.C.

### Vaccination in Switzerland.

The vaccination question is always with us, and those who do not believe in the value of vaccination have thought that evidence to prove their contention was to be had from the experience of Switzerland. In that country twelve of the cantons have a vaccination law which is enforced with more or less strictness, while of the remaining thirteen, two have never passed a vaccination law, nine have repealed a former law, and two, without repealing it, are not enforcing the law which is on their statute-book. With regard to small-pox sickness and mortality in Switzerland, Mr. Runciman recently gave the following information in answer to a question in the House of Commons: Switzerland has not suffered much from small-pox in recent years, but in the five years 1900-4 (inclusive) there have been 800 cases. These cases have been fairly evenly distributed between the cantons which enforce and those which do not enforce a vaccination law. So far the evidence does not seem to favour one theory regarding the value of vaccination more than another. But when analysed a little more closely, a different moral may be drawn. Of the 800 cases, 180 were those of children under ten years of age, and of these children only twenty-six were vaccinated, the remainder being recorded as unvaccinated. This of itself indicates some protective power in vaccination, and the inference is confirmed by the fact that while among the twenty-six vaccinated children there were no deaths, the deaths among the unvaccinated numbered twenty-seven. The value of vaccination cannot be decided merely by reference to law, for, as we know, the same law may be stringently interpreted in one parish and regarded as almost a dead letter in the next. In the absence of more detailed information, therefore, it would be rash to say that vaccination is not an effective protection to adults. But the facts given by Mr. Runciman show that it is a very efficient protection to children under ten.

### Infectious Hospitals in Poor Extensive Rural Districts.

The system of infectious hospitals in rural districts is very unsatisfactory. Where such a so-called hospital exists in a rural district it is frequently found, as was the case in Tunbridge Wells, to be unfit for occupation, or without any staff which could be trusted to take charge of a patient brought in suffering from an infectious disease. Further, there are still many districts, especially poor districts with large population—mining districts, for example, and large quarry works in North Cornwall, where there is no provision of any kind for infectious diseases, and where the local authority has entirely failed to do its duty. This is a poor man's question, and should therefore attract the attention and sympathy of the President of the Local Government Board. It is this department which is responsible to the country for the general inadequacy and inefficiency of so many so-called infectious diseases hospitals, and for the absence of any adequate provision for infectious disease in certain remote rural districts. This is a day of Commissions, and the Local Government Board might bestir itself and justify its existence by the President appointing a small but strong committee to report on the whole question with a view of finding an immediate practical remedy. No doubt the local authorities will deny inefficiency, but the records at the department ought—unless we are without knowledge—to prove much inefficiency and neglect in regard to this type of hospital. The causes are quite simple. During many months they are empty, and it is nobody's business to look after them except perhaps a caretaker, who, when a case arrives, conducts it to the hospital ward, clothes the patient, and, in the absence of a proper nurse, perhaps eats his food in the ward occupied by the patient. It is, to say the least, a mysterious manner of arranging a very important and practieal public duty.

